# Construction of a T-cell exhaustion-related gene signature for predicting prognosis and immune response in hepatocellular carcinoma

**DOI:** 10.18632/aging.204830

**Published:** 2023-06-22

**Authors:** Tianrui Kuang, Lilong Zhang, Dongqi Chai, Chen Chen, Weixing Wang

**Affiliations:** 1 Department of General Surgery, Renmin Hospital of Wuhan University, Wuhan, China; 2 Central Laboratory, Renmin Hospital of Wuhan University, Wuhan, China

**Keywords:** T-cell exhaustion, hepatocellular carcinoma, immunotherapeutic effect, prognosis

## Abstract

Background: Hepatocellular carcinoma (HCC) is a heterogeneous malignancy with a rising prevalence worldwide. Immunotherapy has been shown to improve treatment outcomes for HCC. We aimed to construct a T-cell exhaustion-related gene prognostic model (TEXPM) for HCC and to elucidate the immunologic characteristics and advantages of immunotherapy in T-cell exhaustion-Related Gene-defined HCC groups.

Methods: Single-cell RNA sequencing data were used in conjunction with TCGA Differentially expressed genes (DEGs) to screen for T-cell exhaustion-Related Genes (TEXGs) for subsequent evaluation. Using univariate Cox regression analysis and LASSO regression analysis, five genes (FTL, GZMA, CD14, NPC2, and IER3) were subsequently selected for the construction of a TEXPM. Then, we evaluated the immunologic characteristics and advantages of immunotherapy in groups identified by TEXPM.

Results: The TEXPM was formed with FTL, GZMA, CD14, NPC2, and IER3. The results of the training and validation team studies were consistent, with the low TEXPM group surviving longer than the high TEXPM group (P < 0.001). Multivariate Cox regression analysis demonstrated that TEXPM (HR: 2.347, 95%CI: 1.844-2.987; HR: 2.172, 95% CI: 1.689-2.793) was an independent prognostic variable for HCC patients. The low-TEXPM group was linked to active immunity, less aggressive phenotypes, strong infiltration of CD8+ T cells, CD4 + T cells, and M1 macrophages, and a better response to ICI treatment. A high TEXPM group, on the other hand, was associated with suppressive immunity, more aggressive phenotypes, a significant infiltration of B cells, M0 macrophages, and M2 macrophages, and a reduced response to ICI treatment. FTL is an independent prognostic variable in HCC patients and the knockdown of FTL can affect the biological behavior of hepatocellular carcinoma cells.

Conclusions: TEXPM is a promising prognostic biomarker connected to the immune system. Differentiating immunological and molecular features and predicting patient outcomes may be facilitated by TEXPM grouping. Furthermore, the expression of FTL was found to be an independent prognostic factor for HCC. Knockdown of FTL significantly inhibited proliferation, migration, and invasive activity in liver cancer cells.

## INTRODUCTION

Hepatocellular carcinoma (HCC) is a solid tumor with heterogeneous nature, and its incidence has been increasing year by year in recent years [1, 2]. As prevalent cancer, various factors, including viral infection and cirrhosis, have been shown to contribute to its development [3]. Currently, liver transplantation, immunotherapy, hepatic resection, etc., comprise the majority of HCC’s viable treatment choices. Despite so many systemic treatment options, the prognosis for patients with advanced disease including extrahepatic metastases and invasion remains poor. Combination immunotherapy with anti-programmed cell death ligand 1 (PD-1) and anti-vascular endothelial growth factor antibodies has now replaced surgery as the first-line systemic therapy for advanced HCC, but its objective response rate is only 29.8% [4, 5]. Multiple variables, including the immunological microenvironment (TME), can impact ICI efficacy, and few biomarkers can accurately predict patient prognosis [6]. Individualization of immunotherapy for patients with HCC is possible if relevant prognostic indicators linked with treatment success can be identified [7]. However, we know very little about the TME of HCC, and improved prognostic and therapeutic indicators are required immediately.

Such low objective response rates of immunotherapy for hepatocellular carcinoma may be associated with T-cell exhaustion (TEX). This is because depleted CD8+ T cells (TEX) that induce reduced function are frequently associated with cancer immune escape [8]. T cell exhaustion refers to a broad range of antigen-specific CD8+ T lymphocyte dysfunctional states that were initially characterized in the context of chronic viral infection and occur when these cells persist but are unable to eliminate a pathogenic danger [9, 10]. CD8+ T lymphocytes have an important role in the prognosis of HCC, where oxidative phosphorylated CD8+ T cell subsets have been shown to be a predictor of immunotherapy resistance in HCC patients. When CD8+ T lymphocytes are exhausted, they are unable to play a role in killing tumors [11, 12]. Therefore, we have to focus more on TEX, reversing TEX may be the key to improving the objective response rate of cancers receiving immunotherapy.

The goal of this study is to identify prognostic markers for HCC that can be used to predict the effectiveness of conventional therapies and to suggest the potential of immunotherapies. We investigated the single-cell RNA sequencing (ScRNA-seq) dataset utilizing the Tumor Immune Single-Cell Hub (TISCH) to determine the genes about TEX in HCC [13]. We created the TEX prognostic model in this instance (TEXPM). In addition, we discussed the immunological traits of the TEXPM-defined groups. Finally, we discovered that TEXPM may be able to predict the outcome and success of immunotherapy in patients with HCC. Our analysis shows that TEXPM is a promising prognostic model.

## MATERIALS AND METHODS

### Data source and clinical information

Gene expression and clinical data were retrieved from TCGA (https://portal.gdc.cancer.gov/), the ICGC and the GTEx database (https://xenabrowser.net/datapages/). The raw count data were firstly normalized with transcripts per million (TPM) method and underwent a log2 transformation. Two independent cohorts were employed in our research, with the TCGA-LIHC cohort (tumor: 377) used as a training dataset and the ICGC cohorts (tumor:260) used as a validation dataset. The TEX-related gene were obtained from two scRNA-seq datasets (GSE125449 and GSE140288) in the TISCH (http://tisch.comp-genomics.org/) [13]. P < 0.05 and |log2 FC| ≥ 1 were used as the thresholds for filtering TEX-related DEGs. Meanwhile, the clinical characteristics of TCGA and ICGC cohorts and TEX-related DEGs are presented in Supplementary Table 1.

KEGG and GO research reveal potential roles and pathways of hub genes [14, 15]. Enrichment analyses were performed using the cluster profile package [16]. The visualization module of the cluster profile was used for displaying analysis results. P < 0.05 was selected as the cut-off criterion.

### Construction of risk prognostic model

Univariate Cox analysis of overall survival (OS) was performed to screen TEX-related DEGs with prognostic values. The R package glmnet was used to run LASSO regression analysis to reduce the risk of overfitting and find the appropriate number of TEX-related DEGs involved in model development (TEXPM). The samples were separated into training and test groups to validate the model’s correctness. A risk prognosis model was developed for the training group and the test group.


$$\text{Risk}\;\text{Score}=\sum_{i=1}^{n}\left(\text{TEXPM}\;\text{exp}i\times \text{coef}i\right)$$

where n is the number of OS prognosis TEX-related DEGs, I is the ith TEX-related DEG, and coef is the regression coefficient; the expression of OS prognosis TEX-related DEGs is multiplied by the corresponding regression coefficient and summed to generate the sample risk score [17]. According to the median risk score, samples from the entire sample, training, and test groups were separated into high- and low-risk groups.

### Validation of risk prognostic model

Risk curve analysis, survival analysis, etc. were all used in the risk prognostic models for the training and test groups. The risk prognosis model’s survival status map and risk heatmap were created using R, and the OS prognosis TEXPM and patient survival times were compared between high- and low-risk groups. The R package associated with it is used to plot ROC curves, while the survival and survminer programs are used to build survival curves. Using univariate and multivariate COX regression models, independent prognostic testing was carried out using the survival package in R to see whether the risk score can be used as an independent prognostic factor.

### Estimating of immune cell infiltration (ICI)

In order to estimate the immune score, stromal score, and 22 different types of ICI, the R packages “ESTIMATE” [18] and “CIBERSORT” [19] were used. Analysis was done on the relationship between the ICI components. The R package “ConsensusClusterPlus” [20] then performed a hierarchical agglomerative cluster according to the ICI pattern. The algorithm used by “ConsensusClusterPlus” establishes the cluster count and membership based on stability data from the unsupervised analysis. To guarantee the stability of clustering, this algorithm was run 1000 times.

### Cell culture and siRNA treatment

The American Type Culture Collection (ATCC) provided human HCC cell lines (including HUH7 and HLF). Cells were cultured with DMEM containing 10% fetal bovine serum (FBS). 2 mM L-glutamine, and 100 U/ml penicillin-streptomycin solution. GeneChem (Shanghai, China) produced FTL siRNAs, which were then transfected into cells using Lipofectamine 2000 (Invitrogen, CA, USA) per the manufacturer’s instructions. The sequence of Si-FTL is: sense 5’- GGCGAGUAUCUCUUCGAAA-dTdT-3’ and antisense 5’- UUUCGAAGAGAUACUCGCC TdTd-3’. The cells were grown in DMEM medium with FBS and penicillin-streptomycin for 6-8 hours before the media was changed.

### CCK8 and transwell assay

HUH7 and HLF cells were grown in 96-well plates (3000 cells/plate in 200 ml DMEM) after transfection with FTL siRNA for 48 hours. At 0, 24, 48, and 72 hours, the proliferative ability of the treated cells was discovered. According to the kit’s instructions, Cell Counting Kit-8 (CCK8) reagent (Yeasen, Shanghai, China) was applied to each plate. A microplate spectrophotometer then measured the OD450 value (Thermo Fisher Scientific, MA, USA). To assess the ability of the cells to migrate, HUH7 and HLF cells were transfected with FTL siRNA for 48 hours and grown in 24-well culture plates with membrane inserts having 8 mm pores. The bottom chamber received DMEM (Gibco, NY, USA) supplemented with 10% fetal bovine serum, whereas the top chamber received serum-free DMEM. With a light microscope, numbered. Three times each experiment was carried out.

### Immunohistochemistry

20 pairs of hepatocellular carcinoma tissue and paraneoplastic tissue from a patient undergoing surgery at the Department of Hepatobiliary Surgery, Wuhan University People’s Hospital (Wuhan, China). The expression of FTL was evaluated using immunohistochemistry (IHC). To enhance antigen retrieval, paraffin-embedded tissue sections (5 m thick) were briefly deparaffinized, rehydrated, and treated for 15 min at 100° C with a 10 mM citric acid buffer. Following overnight incubation at 4° C with primary antibodies against FTL (Abclonal, A11241), the sections were then rinsed with PBS and incubated for 30 minutes at 37° C with a corresponding secondary antibody. Sections were stained with hematoxylin and counterstained with DAB following a second PBS wash.

### Mutation and drug-sensitivity analysis

The mutation annotation format (MAF) from the TCGA-LIHC was generated using the “maftools” R package to identify the difference in somatic mutations of HCC patients between high- and low-TEXPM groups [21, 22]. To study the differences in the sensitivity of chemotherapeutic agents commonly used to treat HCC between the two groups, we employed the “pRRophetic” package to calculate the semi-inhibitory 146 concentration (IC50) values.

### Statistical analysis

All statistical analyses and visualizations were carried out utilizing R software version 3.6.3 (https://www.r-project.org/). In the analysis of gene expression between tumor tissues and adjacent nontumorous tissues, a Student’s t-test was employed. The Chi-squared test was utilized to assess differences in proportions. To compare the ssGSEA scores of immune cells or pathways between the high-risk and low-risk groups, the Mann-Whitney test was performed, with P values adjusted using the Benjamini-Hochberg (BH) method. The comparison of OS between different groups was conducted using Kaplan-Meier analysis with the log-rank test. Independent predictors of OS were identified through univariate and multivariate Cox regression analyses. Unless otherwise specified, a P value less than 0.05 was considered statistically significant, and all P values were two-tailed.

### Availability of data and materials

The data that support the findings of this study are available from the corresponding author upon reasonable request.

## RESULTS

### Identification of DEGs associated with TEX in hepatocellular carcinoma

**Figure 1:**
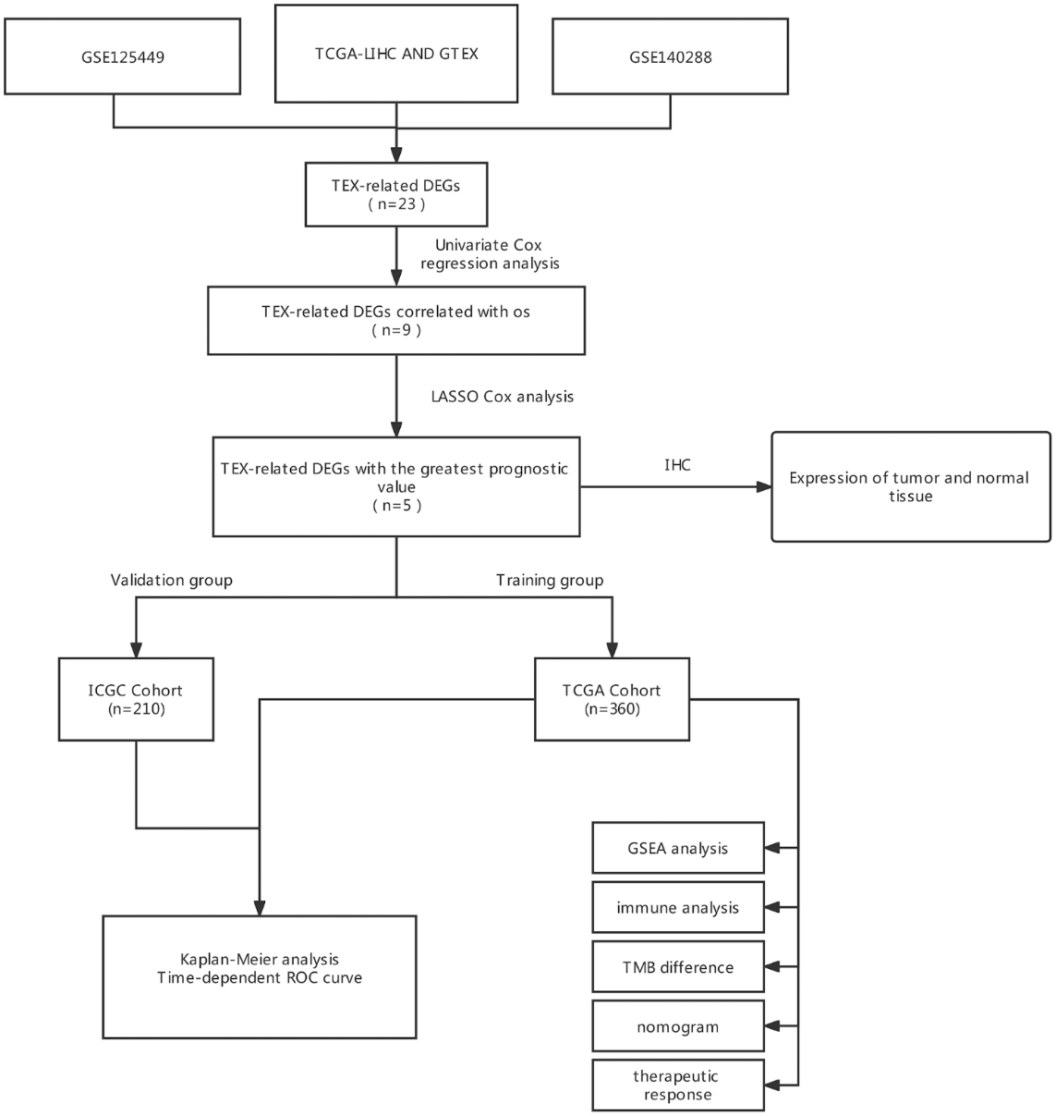
**The flow diagram of our study.** DEGs, differently expressed genes; LASSO, least absolute shrinkage, and selection operator; ROC, receiver operating characteristic; OS, overall survival.

**Figure 2:**
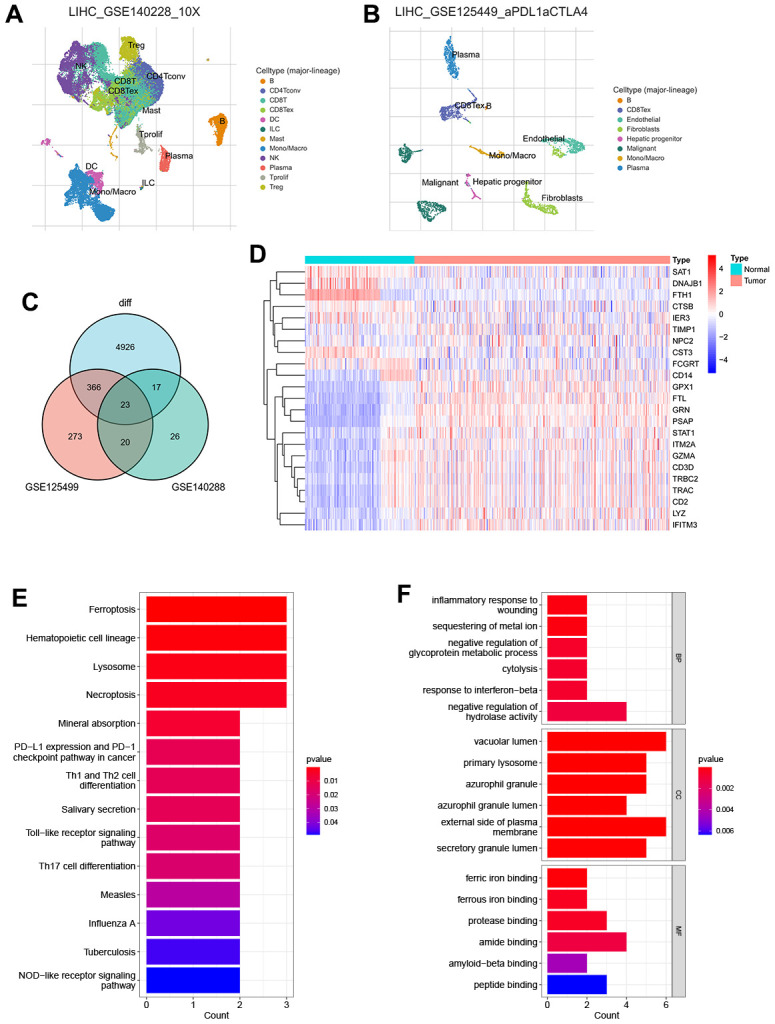
Recognition of TEX-related DEGs (**A**) The UMAP plot in GSE125449 (**B**) The UMAP plot in GSE140228 (**C**) Venn plot of overlap TEX-related DEGs in the three datasets (**D**) Expression heat map of malignant and non-malignant cell markers in TCGA and GTEx databases. (**E**) The bar plot showing TEX-related DEGs by KEGG biological process. (**F**) The bar plot showing TEX-related DEGs by GO biological process.

The general study workflow is depicted in Figure 1. As a tumor microenvironment (TME)-focused scRNA-seq database, TISCH provides valuable resources for TME research. In this study, we utilized TISCH to extract approximately 26 and 273 genes associated with TEX from two single-cell datasets (GSE125449 and GSE140288) (Figure 2A, 2B). Subsequently, we combined these findings with data from the TCGA and GTEx databases, resulting in the identification of 23 TEX-related DEGs (LYZ, FCGRT, STAT1, GRN, PSAP, DNAJB1, CD3D, FTL, GPX1, GZMA, CTSB, SAT1, TRBC2, TRAC, CD14, NPC2, FTH1, IER3, CD2, IFITM3, ITM2A, CST3, TIMP1; Figure 2C). The expression level of TEX-related DEGs was shown by heatmap (Figure 2D). Biological functional analyses revealed that these genes were abundant in the “Ferroptosis,” “PD-L1 expression and PD-1 checkpoint pathway in cancer,” “Th1 and Th2 cell differentiation,” etc., pathways (Figure 2E, 2F), demonstrating the plausibility of our gene set. This established the basis for modeling.

### Construction and validation of TEXPM

**Figure 3:**
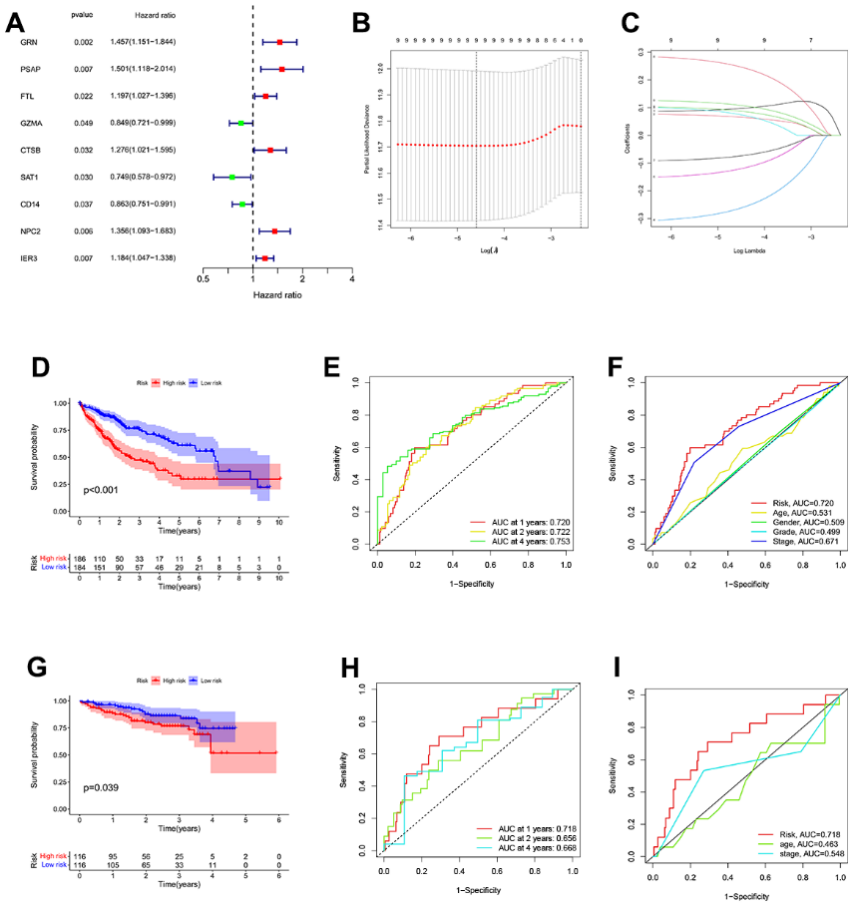
**Construction of a TEX-related DEGs signature for prognosis.** (**A**) 9 TEX-related DEGs show remarkable relevance to OS according to univariate Cox regression analysis. (**B**, **C**) The LASSO coefficient profiles were constructed from 9 prognostic TEX-related DEGs, and the tuning parameter (λ) was calculated based on the minimum criteria for OS with ten-fold cross-validation. five genes were selected according to the best fit profile. (**D**) Kaplan-Meier survival analysis of high- and low-TEXPM groups in the TCGA cohort. (**E**) ROC analysis for OS prediction including 1, 2, and 4 years of HCC patients in the TCGA cohort. (**F**) ROC curve analysis compares the predictive power of the MRS signature and other clinicopathological indicators in the TCGA cohort. (**G**) Kaplan-Meier survival analysis of high- and low-TEXPM groups in the ICGC cohort. (**H**) ROC analysis for OS prediction including 1, 2, and 4 years of HCC patients in the ICGC cohort. (**I**) OC curve analysis compares the predictive power of the MRS signature and other clinicopathological indicators in the ICGC cohort.

To enhance the precision of the prognostic model, we include the TCGA and GTEx cohorts in the training team and the ICGC Cohort in the test team. The univariate COX analysis found nine important TEX-related genes, and nine genes in total were identified as independent HCC prognostic indicators (Figure 3A).

Additional research, multivariate Cox regression analysis, and LASSO regression revealed that five genes (FTL, GZMA, CD14, NPC2, and IER3) were prognostic markers that were used to construct a TEXPM (Figure 3B, 3C). On the training and test team, the prognostic performance of the TEXPM was validated using the KM survival curve and log-rank test. On the basis of the median risk score, each case was classified as either high- or low-group. Based on KM analysis, the presence of the high-TEXPM group was associated with a lower likelihood of survival in the TCGA and GTEx cohorts (P < 0.001, Figure 3D). For1, 2 and 4- year survival rates, the AUC predictive value of the TEXPM was 0.720, 0.722 and 0.753 (Figure 3E). Furthermore, the AUC value for TEXPM was substantially greater than those for age, sex, tumor stage, and pathological stage (Figure 3F). Similar to the TCGA findings, the majority of new TEXPM identified in this analysis were negatively associated with the risk model in the test group. (P = 0.0039; Figure 3G) The existence of a high- TEXPM group was related to a decreased likelihood of survival. The AUC predictive value of the TEXPM for 1, 2 and 4- year survival rates was 0.718, 0.661 and 0.669 (Figure 3H).

After we determined the optimal risk score cutoff point, TCGA and ICGC patients were divided into high and low TEXPM groups. Using a prognosis curve and a scatter plot, the risk score and survival status of each HCC patient were determined (Supplementary Figure 1). In addition, the majority of deaths were concentrated in the high-TEXPM group (Supplementary Figure 1). In addition, the heat map of candidate DEGs’ expression patterns revealed that FTL, NPC2, and IER3 were strongly expressed in the high-TEXPM group, whereas GZMA and CD14 were substantially expressed in the low-TEXPM group (Supplementary Figure 1). Collectively, these data identified five TEX-related DEGs as the characteristic prognostic marker for HCC patients.

### Independent prognostic analysis of TEXPM

**Figure 4:**
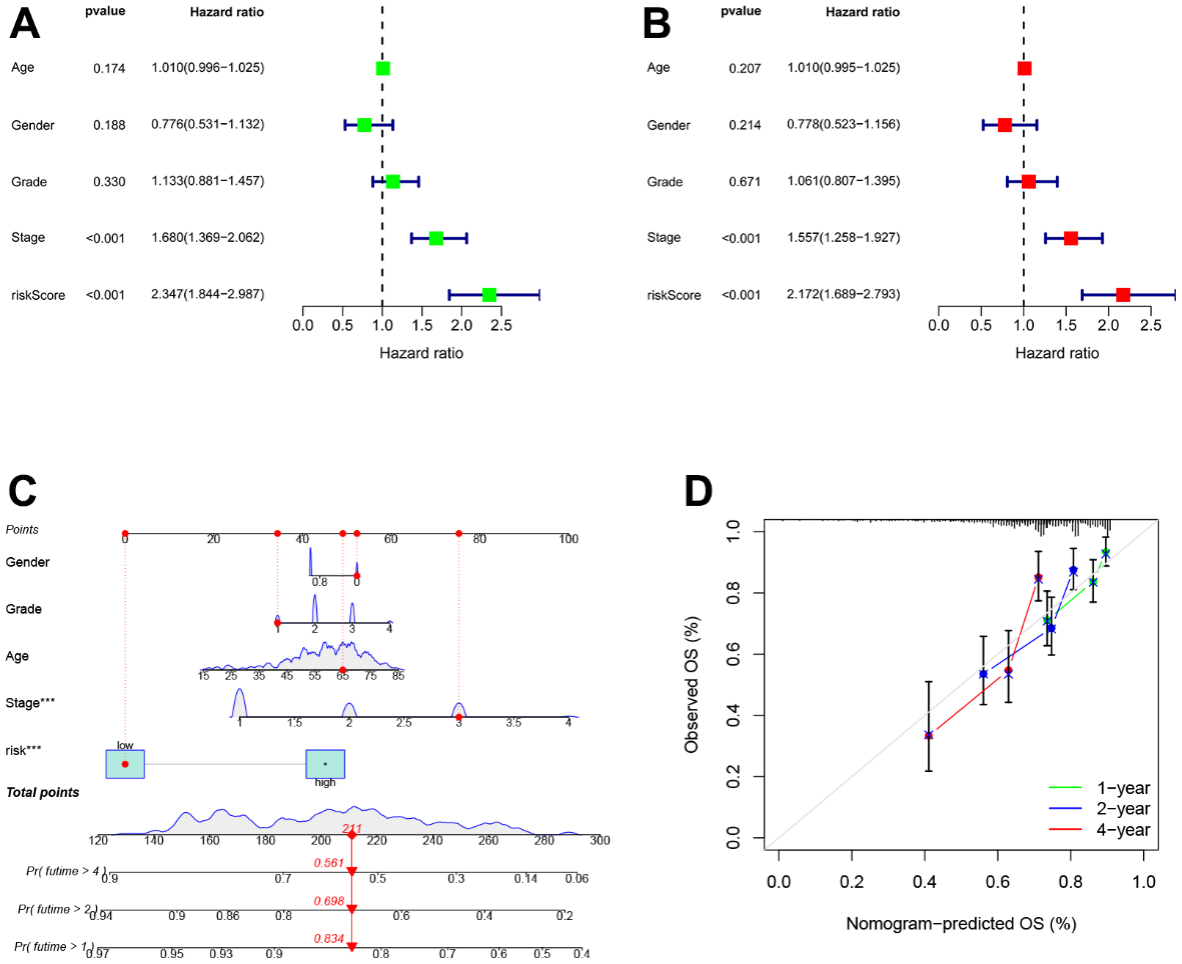
**Establishment and assessment of the nomogram for survival prediction.** (**A**, **B**) Univariate and multivariate Cox regression analyses showed that risk score based on TEXPM is an independent prognostic factor affecting the prognosis of HCC patients. (**C**) The nomogram combining risk score based on TEXPM was developed to predict 1-, 2-, and 4-year survival. (**D**) Calibration curves show the predictions of the nomogram that we established for 1-, 2-, and 4-year overall survival.

Next, univariate Cox analysis demonstrated that TEXPM (HR: 2.347, 95% CI: 1.844-2.987) and stage (HR: 1.679, 95% CI: 1.368-2.061) were independent prognostic variables for HCC patients (Figure 4A). Both the TEXPM (HR: 2.172, 95% CI: 1.689-2.793) and the stage (HR: 1.557, 95% CI: 1.258-1.927) were independent prognostic risk factors for HCC patients, according to the multivariate Cox analysis (Figure 4B). To make the TEXPM more useful in the clinic, a nomogram was developed to investigate the TEXPM’s capacity to predict 1-, 2-, and 4-year survival in the TCGA and GTEx cohorts. As depicted in Figure 4C, the nomogram’s predictive criteria included the innovative risk score model and additional clinicopathological characteristics. Similar to the results of the multivariable Cox regression analysis, it was determined that the risk score model had the most weight in this combined nomogram among all these clinically important factors. Collectively, these investigations have shown that TEXPM might dependably serve as an independent predictive indicator for HCC patients.

### Molecular features in high- and low-TEXPM groups

**Figure 5:**
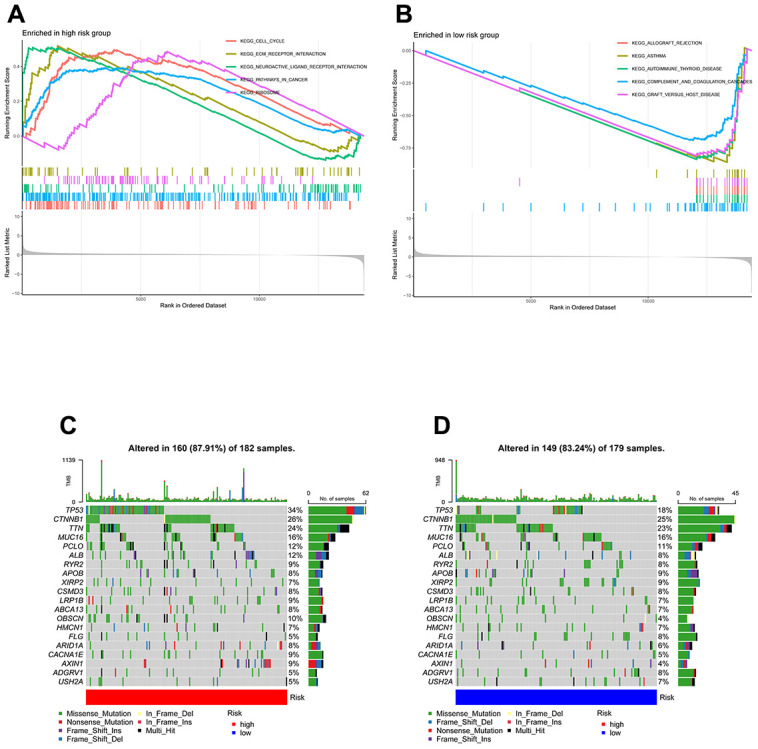
**GSEA and mutation in high- and low-TEXPM groups.** (**A**) GO gene sets enriched in the high-TEXPM group. (**B**) GO gene sets enriched in the low-TEXPM group. (**C**, **D**) Significantly mutated genes in the mutated HCC samples of different TEXPM groups. Mutated genes (rows, top 10) are ordered by mutation rate; samples (columns) are arranged to emphasize mutual exclusivity among mutations. The right shows the mutation percentage, and the top shows the overall number of mutations. The color coding indicates the mutation type.

GSEA was used to identify enriched GO gene sets in the two TEXPM groups, detailed GSEA results are in Supplementary Table 2. The GSEA plot only showed the top five routes. The gene sets of the low-TEXPM group were enriched in immune-related self-limiting disease pathways, whereas the high-TEXPM group was enriched in cell cycle and cancer-related pathways (Figure 5A, 5B). When the high- and low-TEXPM groups were analyzed for gene mutations, we found that TP53 accounted for the largest proportion and that TP53 was more frequent in the high-TEXPM group than in the low-EXPM group (Figure 5C, 5D). As shown in Supplementary Figure 2. we analyzed the correlation between the TEXPM score and TMB. Although not statistically significant (r = 0.077, P = 0.15), Kaplan-Meier analysis showed that the presence of high-risk TEXPM and high TMB groups was associated with a reduced likelihood of survival (P < 0.001).

### Immune characteristics of high- and low-TEXPM groups

**Figure 6:**
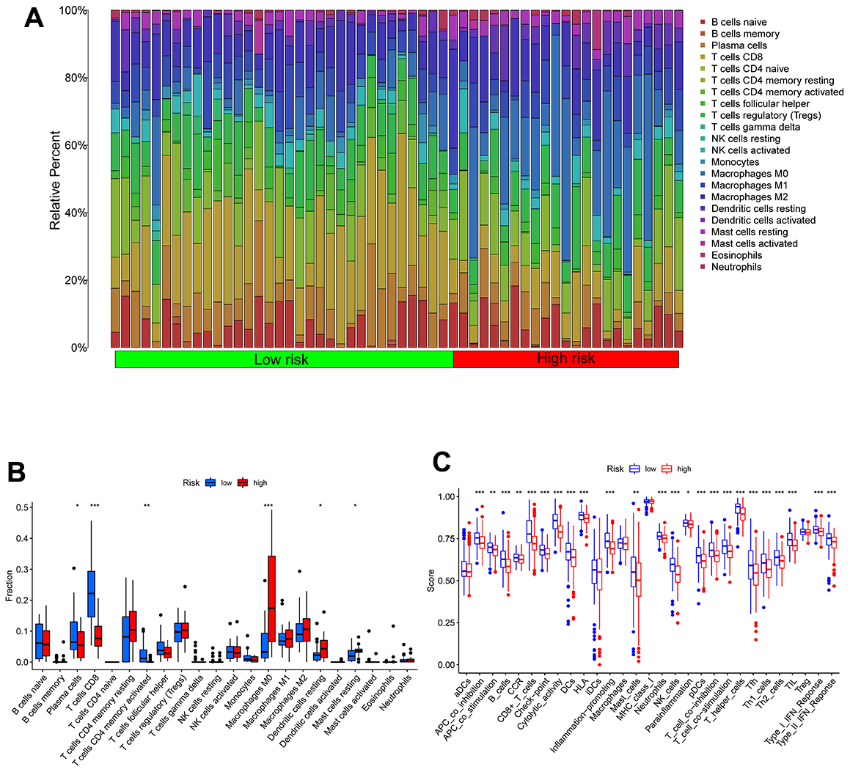
**Immune characteristics in high- and low-TEXPM groups.** (**A**, **B**) The proportions of TME cells in high- and low-TEXPM groups. (**C**) The molecular and immune-related function in high- and low-TEXPM groups. (**p < 0.01, ***p < 0.001).

We utilized the Wilcoxon test to evaluate the distribution of immune cells in different TEXPM groups in order to analyze the composition of immune cells in different TEXPM groups. In the low-TEXPM group, there were more activated memory CD4+ T cells, M1 macrophages (anti-tumor phenotype), and CD8+ T cells, while the high-TEXPM group had more M2 macrophages (pro-tumor phenotype) (Figure 6A, 6B). Then, using specific gene signatures, we distinguished the immunological and molecular functions between the two groups. As a result, the low-TEXPM group’s immunological and molecular functions were more active (Figure 6C). As shown in Supplementary Figure 3, The TEXPM was negatively correlated with most checkpoints such as TIGIT and LAG3. The several TEXPM genes, including GZMA and NPC2, showed positive correlations with almost all checkpoints.

**Figure 7:**
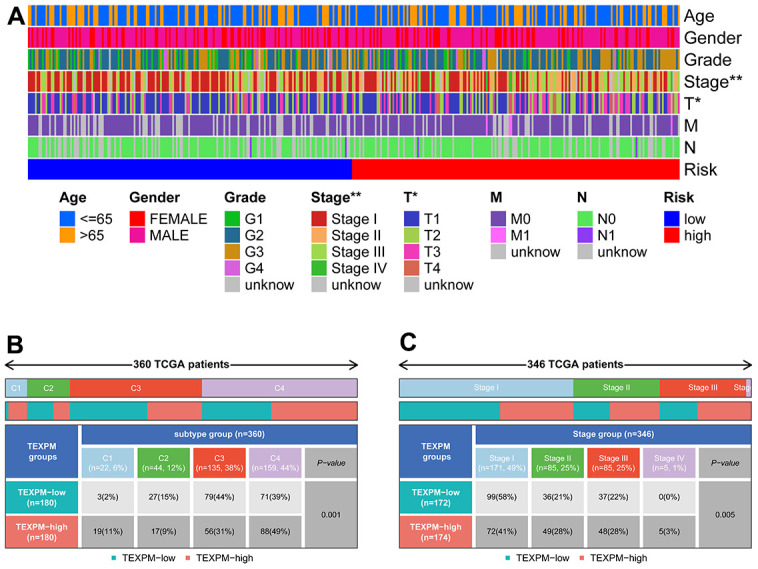
**Relationship between TEXPM and clinical subtypes.** (**A**) The TEXPM groups and clinical subtypes for HCC patients in the TCGA cohort. Age, gender, tumor grade, and TNM stage are shown as patient annotations. (**B**) Heat map showing the distribution of immune grade (C1-4) between high- and low-CDIGPM groups. (**C**) Heat map showing the distribution of HCC TNM stages (stage 0-IV) between high- and low- TEXPM groups. (***p < 0.001).

Figure 7A shows the clinical features in high- and low-TEXPM groups. In Figure 7B, the high TEXPM group was more predominant at the level of immune subtype C1 and immune subtype C4, while the low TEXPM group was more predominant at the level of immune subtype C2 and immune subtype C3 (P = 0.001). In Figure 7C, Specifically, In Stage I, the low-TEXPM group accounted for 58% of the total low-TEXPM group, while in Stages II, III, and IV, high-TEXPM groups totaled 59% of the total high-TEXPM group (P = 0.005).

### Correlation between TEXPM and immunophenoscore (IPS) analysis and drug sensitivity

**Figure 8:**
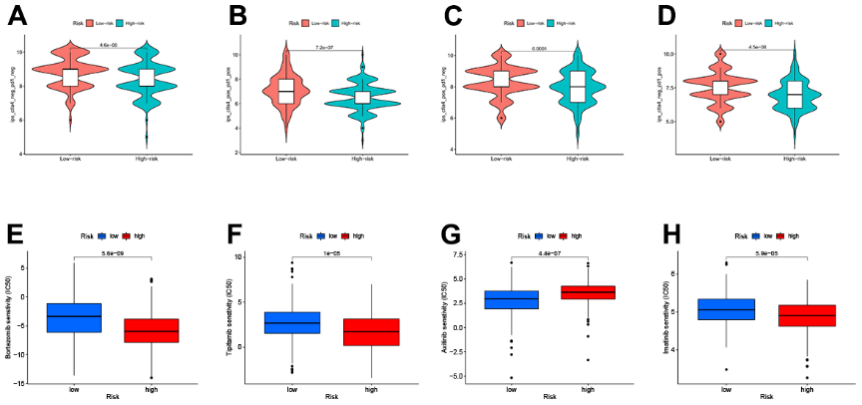
Relationship between TEXPM and (IPS) analysis and drug sensitivity (**A**–**D**) low-TEXPM and high-TEXPM response to IPS. (**E**–**H**) Treatment of different small molecule drugs by high and low-TEXPM groups.

The IPS file received from TCIA was utilized to see if TEXPM expression may predict the immunotherapy response of HCC patients [23]. The IPS was obviously greater in the low-TEXPM group, indicating that immunotherapy would be more effective in patients with the low-TEXPM group (Figure 8A–8D). Next, we selected chemotherapeutic agents, reviewing the drugs currently used to treat LIHC to assess the sensitivity of these drugs in both groups of patients. We found that IC50 values for Imatinib, Bortezomib, and Tipifarnib were lower in patients with low TEXPM, while IC50 values for Axitinib were significantly lower in patients with high TEXPM (Figure 8E–8H).

### Validation of the role of FTL in HCC cells

**Figure 9:**
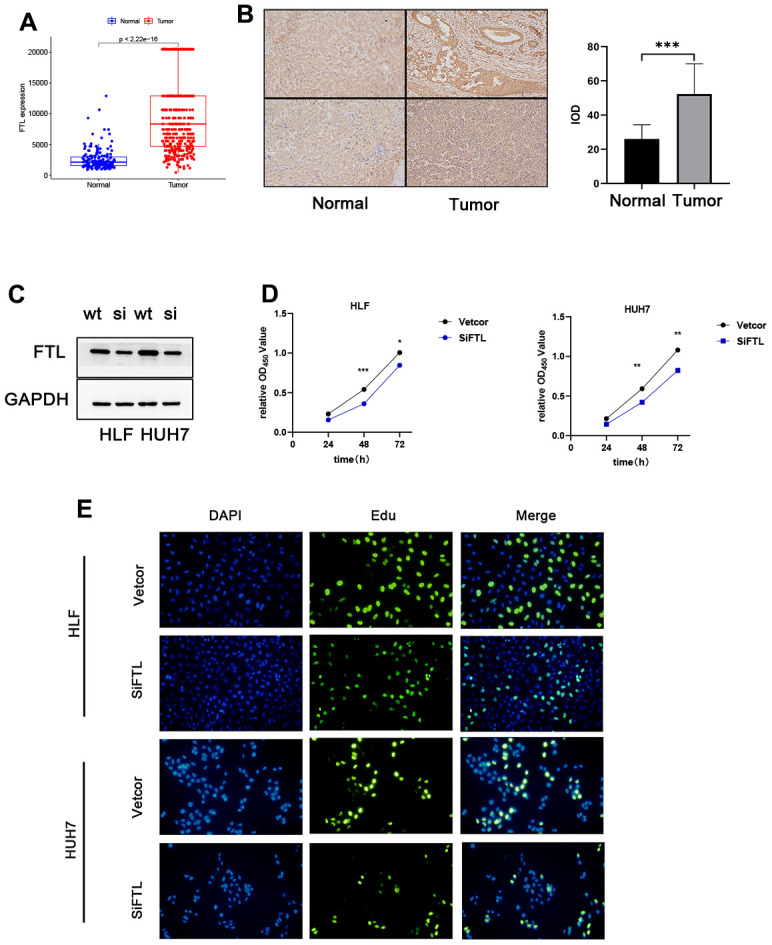
**FTL promoted proliferation in HCC.** (**A**) TCGA database analysis indicated that FTL mRNA expression is increased in HCC. (**B**) Immunohistochemical results suggest that FTL is highly expressed in tumor tissues. (**C**) Western blot analysis confirmed that the expression of FTL was inhibited by SIRNA. (**D**) CCK8 assay indicated that FTL inhibition significantly suppressed the proliferation in HCC cells. (**E**) EdU assays indicated that FTL inhibition significantly suppressed the proliferation in HCC cells. *P < 0.05, **P < 0.01, ***P < 0.001.

**Figure 10:**
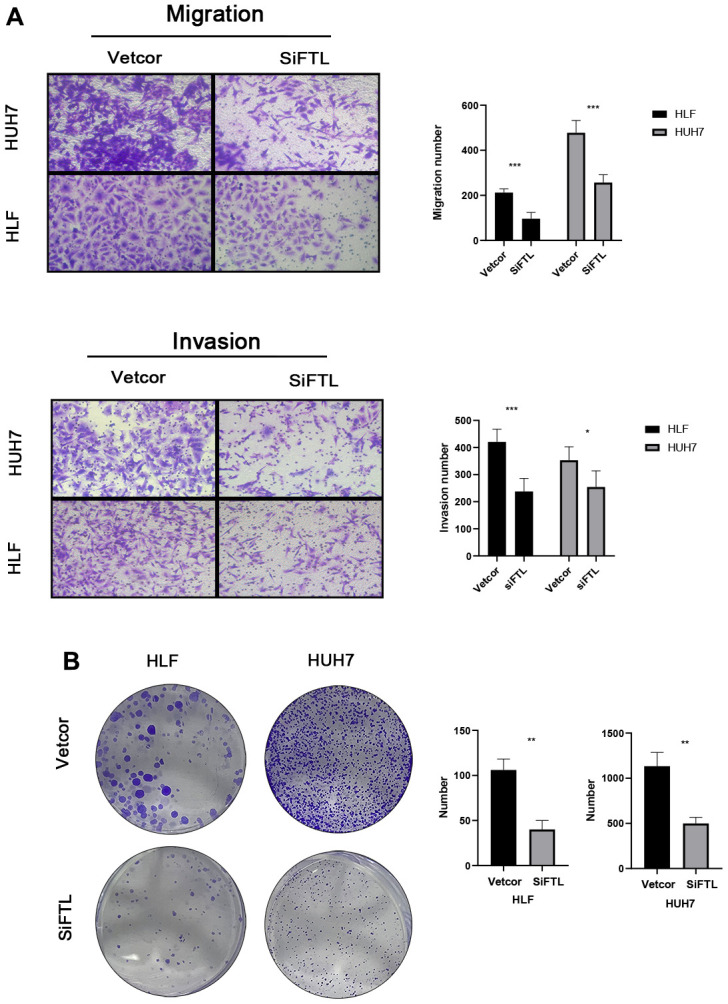
FTL promoted EMT in HCC (**A**) Transwell assays confirmed that FTL inhibition inhibited the migration and invasion of HCC cells. (**B**) Knockdown of FTL reduced colony numbers in HCC cells. *P < 0.05, **P < 0.01, ***P < 0.001.

Using the TCGA and GTEx data, we could learn the expression of five genes of the TEXPM construct, FTL, GZMA, CD14, and NPC2 were highly expressed in tumor tissues, and IER3 was highly expressed in paracancerous tissues (Supplementary Figure 4). To investigate further the role of FTL in HCC cell proliferation and migration. Immunohistochemistry revealed that FTL is abundantly expressed in hepatocellular cancer tissues (Figure 9A). We decreased the amount of FTL in HCC cell lines (including HUH7 and HLF cells). Western blotting showed that treatment with FTL siRNA substantially inhibited FTL expression in HUH7 and HLF cells (Figure 9C). The CCK test revealed that FTL inhibition greatly suppressed HCC cell growth (Figure 9D). In addition, Edu assays were able to confirm the effect of FTL on proliferation in HCC cells (Figure 9E). Moreover, transwell study results demonstrated that FTL reduced the migration and invasion of HUH7 and HLF cells (Figure 10A). Cell colony formation experiments showed that FTL knockdown greatly reduced the number of colonies in HUH7 and HLF cells (Figure 10B). In conclusion, FTL could promote the proliferation, migration and invasion of HCC cells.

## DISCUSSION

Immunotherapy has been acknowledged as the primary treatment for HCC patients by guidelines [4, 24, 25]. Since the objective remission rate of immunotherapy for liver cancer remains low, we currently need to find exactly the patients who benefit. After years of testing many prognostic markers in HCC, there is currently no confirmed biomarker for predicting immunotherapy response and OS. This emphasizes the necessity to establish a biomarker for immunotherapy prognosis in HCC.

As the main anti-tumor cell, T cells have the ability to actively attack tumor tissue [26]. From Tisch, we selected genes related to TEX by analyzing two scRNA-seq datasets (GSE125449 and GSE140288). Subsequently, we merged the TEX-related genes with the differentially expressed genes obtained from the TCGA dataset, resulting in the identification of 23 TEX-related DEGs. Next, we performed univariate COX regression analysis with the 23 genes and obtained 9 differential genes associated with prognosis. After that, we performed LASSO Cox regression analysis to reduce the number of TEX-related DEGs in the risk model and finally obtained TEXPM consisting of five genes. Using TCGA and ICGC arrays, in addition to being more accurate in predicting outcomes than other clinical risk indicators (grade, stage, and age), TEXPM is also able to differentiate HCC patients into two groups based on OS.

Then, to gain more insight into the immunological properties of TEXPM, we examined the genetic mutations in various TEXPM populations. As previously reported, missense variants were the most prevalent, followed by nonsense and frameshift deletions [27]. TP53 mutations, which were more prevalent in the high-TEXPM group than the low-TEXPM group (34% vs. 18%), exhibited the highest variation in mutation frequency between groups. Not only are TP53 mutations prevalently inherited in cancer, but they also result in aggressive malignancies and a worse prognosis for patients [28, 29]. Through the p53/TGF-b signaling pathway, TP53 can affect the cancer cell cycle. Additionally, the TEXPM-high group had a greater rate of PIK3CA mutation than the TEXPM-low group, which may indicate that TEXPM-high HCC promotes proliferation via the PI3K-AKT signaling pathway [30]. Accordingly, our survival data are consistent with the fact that patients with high TEXPM levels who also have high TP53 and PIK3CA mutations will fare worse than those with low TEXPM levels who also have low TP53 and PIK3CA mutations.

Finally, a better understanding of TME may help to develop new treatments for HCC or repair of TME to improve the effectiveness of immunotherapy. Some immune cells were composed differently between the two TEXPM groups. B cells and M0 and M2 macrophages were more prevalent in the high-TEXPM group, whereas cytotoxic CD8 T cells, CD4 T cells, and M1 macrophages were more abundant in the low-TEXPM group. Numerous studies have shown that a dense infiltration of T cells, particularly cytotoxic CD8 T cells, is a sign of good prognosis [31–33]. M2 macrophages, a subtype of macrophages, are linked with chronic inflammation and have a role in stimulating tumor development in the majority of the tumors we discovered. Moreover, these cells have been linked to a poor prognosis in malignancies such as breast, bladder, and ovarian [32, 34, 35]. In contrast, the high infiltration of M1 macrophages may be associated with acute inflammation, which has been reported to suggest a positive prognosis for HCC patients [35]. Our study’s findings support these conclusions. Moreover, based on the results of pathway enrichment, we discovered that the low-TEXPM group had a greater capacity for damage repair, whereas the high-TEXPM group contained more immunosuppressive cells and signals, as well as tumor- and metastasis-related signals, indicating that the high- TEXPM group exhibited immunosuppression and active tumor progression.

To define intratumoral immune states, Vesteinn et al. scored 160 immune expression signatures and utilized cluster analysis to identify immune signature modules [36, 37]. Notably, in our research, immunological subtypes C1 and C4 were mostly found in the high-TEXPM group, while immune subtypes C2 and C3 were primarily found in the low-TEXPM group. C1 represents the Wound Healing immune subtype, which is characterized by a high expression of angiogenic genes and a Th2 cell bias. C2 is the immunological subtype with the largest M1 macrophage polarization, CD8 T cell infiltration, and T cell receptor (TCR) diversity. Now we understand that M1 macrophages play a significant role in pro-inflammatory responses, whereas M2 macrophages play the opposite role. C4, a lymphocyte-deficient phenotype, displayed a more prominent macrophage signature, with Th1 suppressed and high M2 response. Strong evidence suggests a connection between increased adaptive immune and CD8 T cell infiltration in malignancies. TCR diversity was also found to have a favorable correlation with overall survival in breast cancer patients [36]. Maybe all these features contribute to the relatively decreasing risk of the low-TEXPM group.

It has been demonstrated in HCC patients that IPS data downloaded from the TCIA provides a prediction score for assessing a patient’s immune therapy response [38–40]. IPS was greater in the group with low- TEXPM, suggesting that persons with low -TEXPM may have a more favorable response to ICI therapy. This study reveals that TEXPM, which has not been previously examined in HCC, may have a strong correlation with the immune infiltration of HCC, indicating the potential relevance of TEXPM in assessing immunotherapy response. In early-stage HCC patients, surgical treatment, ablation, or liver transplantation are all effective treatment modalities that can significantly improve the survival time of patients [4]. For patients with advanced HCC, systemic treatment is the only option to improve survival. In addition to the use of immunotherapy-related drugs, we tend to use some chemotherapy drugs as well, and in the vast majority of them, the low-TEXPM group will have a better treatment effect than high-TEXPM, thus improving the survival time of HCC patients.

Based on the above findings, we otherwise conclude that TEXPM is a good model to predict survival time in HCC patients and is also closely related to the immune microenvironment. An in-depth study of TEXPM would be beneficial to reverse T cell depletion and thus improve the efficacy of immunotherapy. Next, five genomes make up TEXPM: FTL, GZMA, CD14, NPC2, and IER3. Ferritin Light Chain (FTL) is one of the iron metabolism regulators, and FTL has been identified for a very long time as one of the iron metabolism regulators. In recent years, an increasing number of investigations have demonstrated the tight association between FTL and malignant tumors [41–43]. GZMA from cytotoxic lymphocytes cleaves and activates GSDMB in order to cause pyroptosis in target cells [44]. Gao et al. showed that a combined therapy involving the modification of GZMA-F2R communication and the use of an anti-PD-1 antibody would be significantly more effective in treating HCC patients [45]. CD14 is a pattern recognition receptor (PRR) that facilitates innate immune responses. CD14 was initially identified as a monocyte marker that signals intracellular reactions in response to bacterial interactions [46]. The protein-coding gene NPC2 (NPC Intracellular Cholesterol Transporter 2) has a lipid recognition domain and has been connected to the innate immune system and lipoprotein metabolism pathways [47]. The expression of the early response gene immediate early response 3 (IER3) is stimulated by numerous stimuli, including growth hormones, cytokines, ionizing radiation, viral infection, and other forms of cellular stress. IER3 exhibits a paradoxical and complex involvement in cell cycle regulation and apoptosis [48].

Four genes, CD14, NPC2, IER3, and GZMA, have been extensively investigated in hepatocellular carcinoma [45, 49–51]. On the other hand, FTL and hepatocellular carcinoma have been less studied. And FTL is able to regulate intracellular ferritin production, a process that may be able to lead to Ferroptosis [52, 53]. Ferroptosis has been investigated as a new form of necrosis in a variety of cancers. The search for its biological targets may be the next generation of cancer treatment. Therefore, we chose FTL for the next step of our study, and immunohistochemistry and the TCGA database verified that FTL is substantially expressed in hepatocellular carcinoma tissues. We discovered that SiRNA knockdown of FTL can reduce the activity of HUH7 and HLF cells, as well as their migration and invasion. All of these experimental data demonstrate that we can impact the course of HCC by interfering in FTL. In addition, we have previously identified FTL as an independent prognostic factor in HCC patients, and in combination with its effect on cell biological behavior, we believe that FTL has the potential to be an emerging therapeutic target for HCC patients. However, our research has several drawbacks. Our Research is primarily based on integrative bioinformatics, and there is yet no experimental validation for these findings. In addition, the accuracy of TEXPM for the prognosis and immune modulation of HCC patients will continue to be a major clinical concern. Specifically, the clinical application guidelines for this predictive risk score model must be clarified.

In conclusion, TEXPM is a promising prognostic biomarker connected to the immune system. Differentiating immunological and molecular features and predicting patient outcomes may be facilitated by TEXPM grouping, TEXPM may be a possible prognostic predictor of immunotherapy. Furthermore, it was observed that the expression of FTL independently served as a prognostic factor for HCC. Notably, the downregulation of FTL resulted in a significant suppression of proliferation, migration, and invasive capabilities in liver cancer cells. we need more studies to confirm the reliability of this model.

## Supplementary Material

Supplementary Figures

Supplementary Table 1

Supplementary Table 2
