# Supplementary material for: Construction of a T-cell exhaustion-related gene signature for predicting prognosis and immune response in hepatocellular carcinoma
Source: Aging (Albany NY). 2023 Jun 22;15(12):5751–74. doi: 10.18632/aging.204830 (PMC10333082; doi:10.18632/aging.204830)
Supplement: Supplementary Figures [file aging-15-204830-s001.pdf]

SUPPLEMENTARY FIGURES

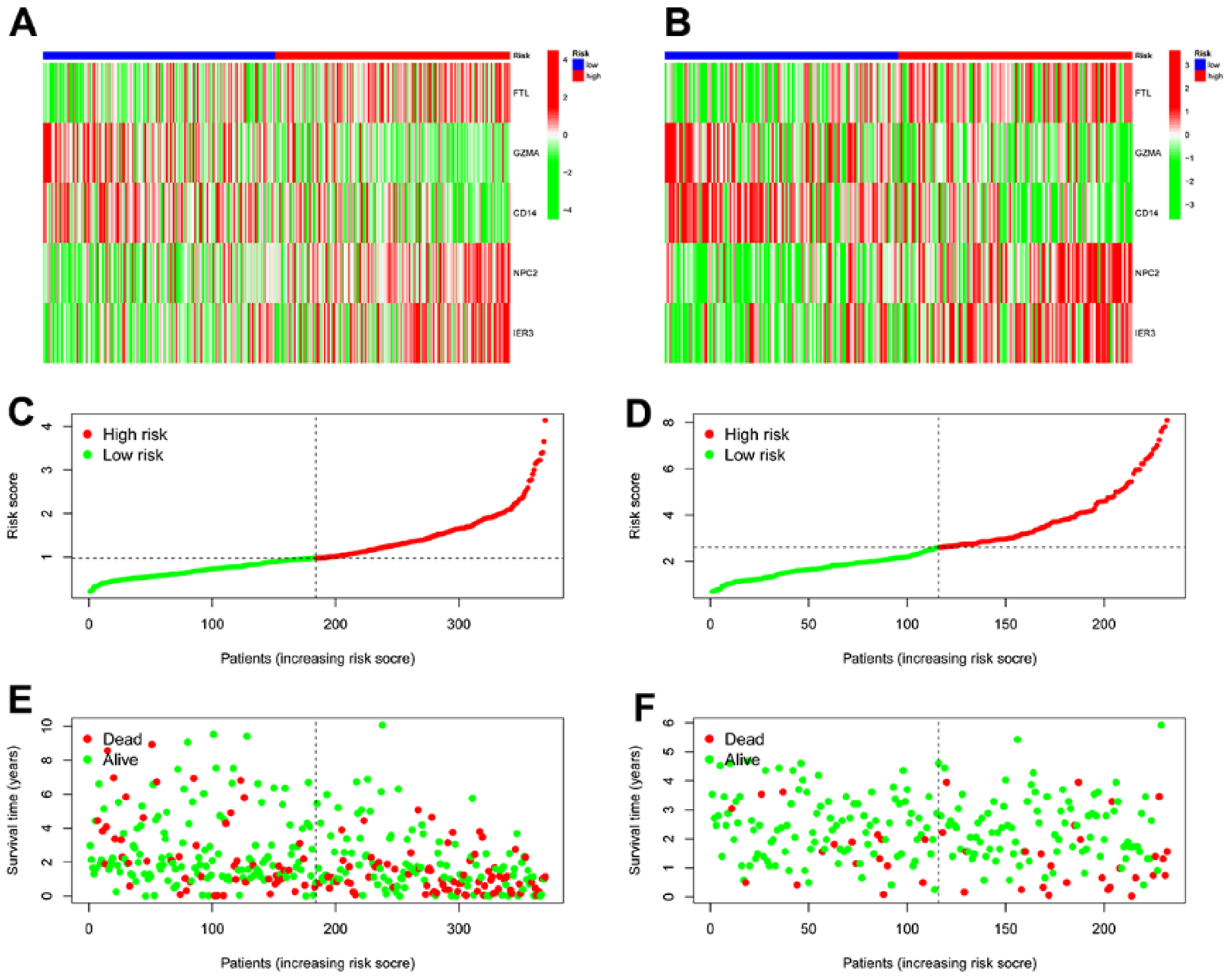

Supplementary Figure 1. Training group and test group. (**A**, **B**) Risk heatmap in the Training group and Test group. (**C**, **D**) Risk score in the Training group and Test group. (**E**, **F**) Survival status map in Training group and Test group.

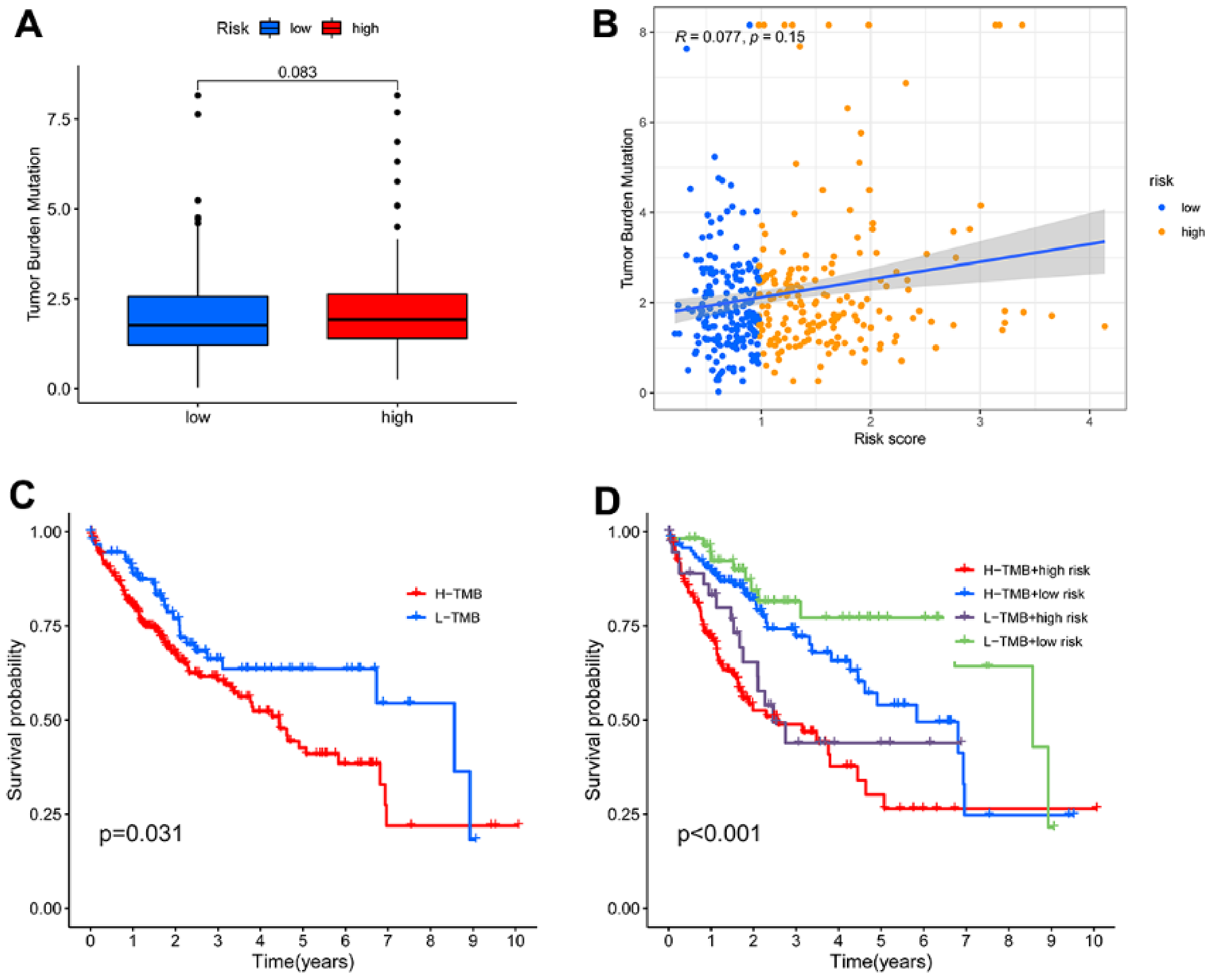

Supplementary Figure 2. The correlation between the TEXPM and TMB. (**A**) The TMB is higher in the high-TEXPM group compared with the low-TEXPM group. (**B**) The correlation between the risk score of TEXPM and TMB. (**C**) OS of the low-TMB group is better than that of the high-TMB group. (**D**) OS of the low-TMB+ low-risk group is better than that of the high-TMB+ high-risk group.

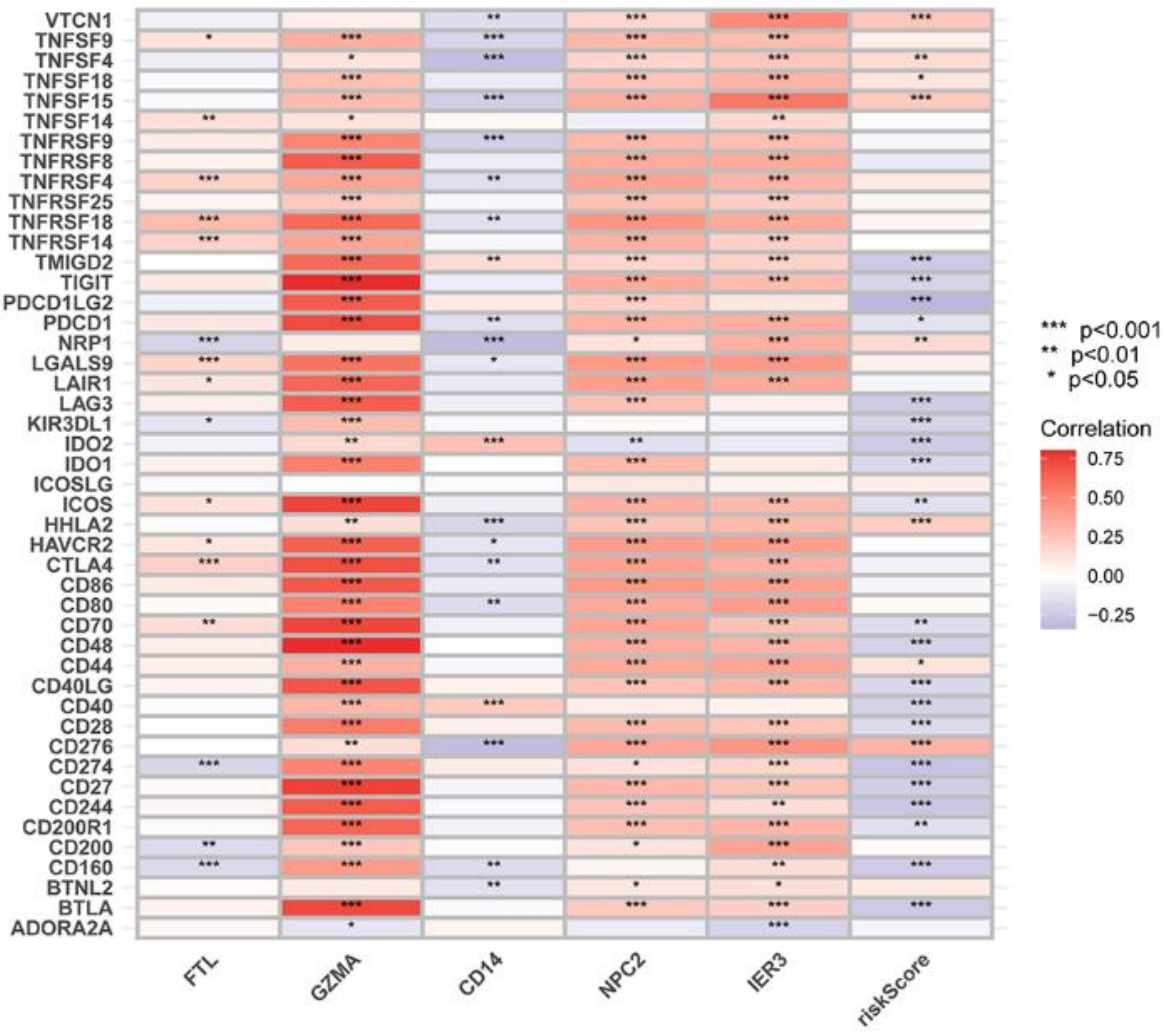

Supplementary Figure 3. Correlation of TEXPM with checkpoints.

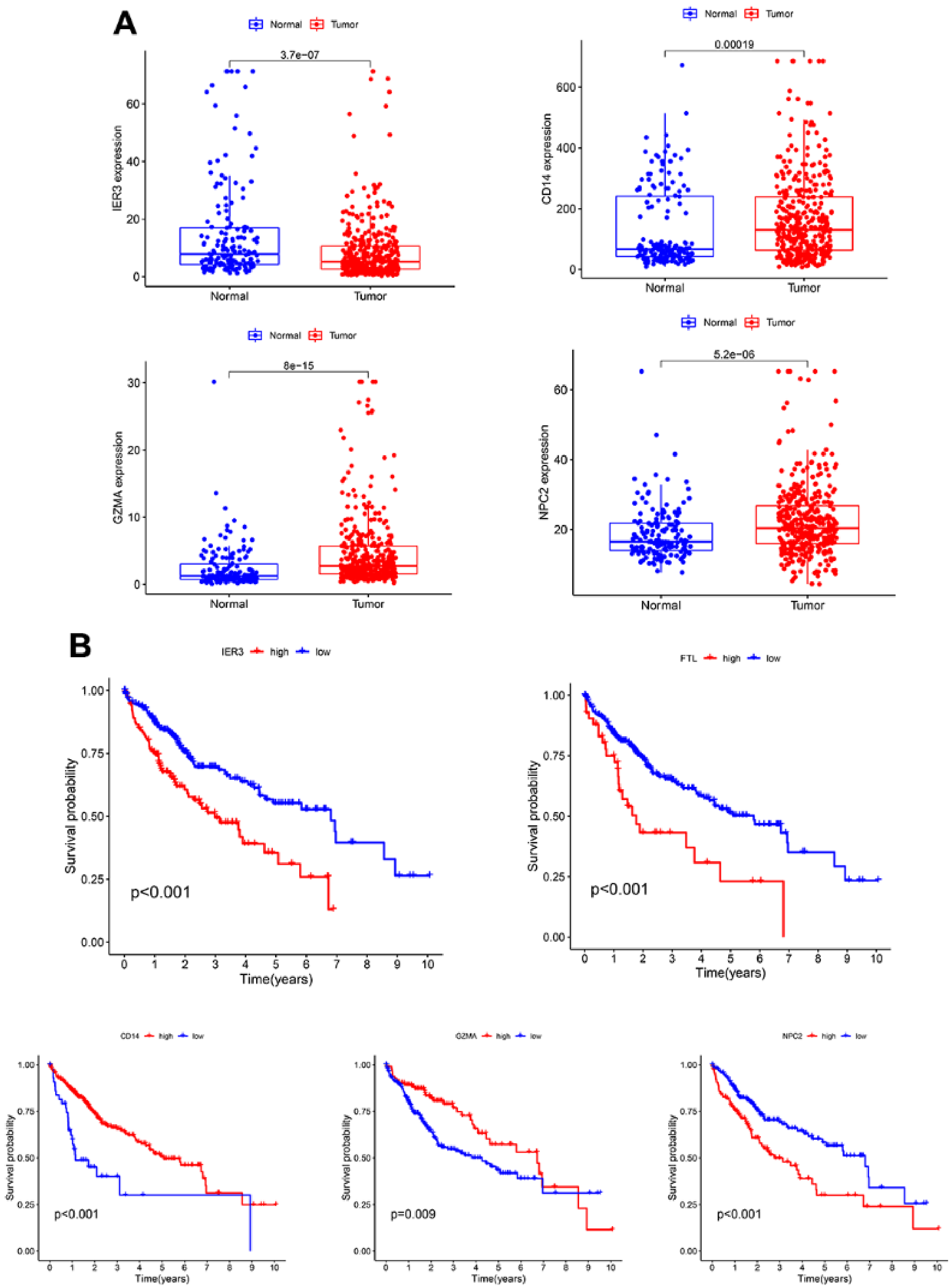

Supplementary Figure 4. Expression of five genes in TEXPM in TCGA. (**A**) The differential expression of 4 genes. (**B**) Survival analysis of 5 genes.
